# Advances in Selective Detection of Cadaverine by Electronic, Optical, and Work Function Sensors Based on Cu-Modified B_12_N_12_ and Al_12_N_12_ Nanocages: A Density Functional Theory (DFT) Study

**DOI:** 10.1021/acs.langmuir.4c02699

**Published:** 2024-10-22

**Authors:** Natanael de Sousa Sousa, Rafael Pereira Silva, Jaldyr de Jesus Gomes Varela Júnior, Adeilton Pereira Maciel

**Affiliations:** † Universidade Federal do Maranhão, 65080-805, São Luís, MA, Brazil

## Abstract

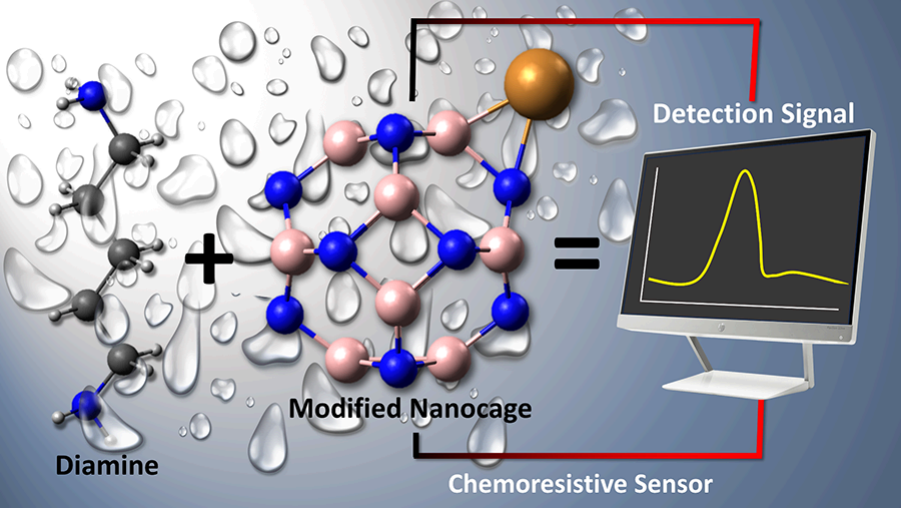

This work explores Cu-modified B_12_N_12_ and Al_12_N_12_ nanocages for cadaverine diamine (Cad) detection using advanced density functional theory (DFT) calculations. The study found that Cu modification altered the geometry of the nanocages, increased the dipole moment, reduced the energy gap, and enhanced the reactivity. While pristine B_12_N_12_ and Al_12_N_12_ were not sensitive to Cad, the modified Cu(b_64_)B_12_N_12_ and Cu(b_66_)Al_12_N_12_ nanocages showed significantly higher electronic sensitivity (Δgap = 39.8% and 35.6%, respectively), surpassing the literature data. However, molecular dynamics (MD) revealed that the Cu(b_66_)Al_12_N_12_ nanocage is not stable in the long term, since the nanocage changes configuration to Cu(b_64_)Al_12_N_12_, which is less sensitive and has an even longer recovery time for Cad sensing. Adsorption energy analysis (*E*_ads_) showed a strong interaction of Cad/nanocages, while charge analysis suggested that the nanocages act as Lewis acids, accepting electrons from Cad. UV–vis spectra confirmed that Cu(b_64_)B_12_N_12_ responds optically to the presence of Cad. Furthermore, Cu(b_64_)B_12_N_12_ showed greater sensitivity to Cad compared to NO, H_2_, H_2_S, CO, COCl_2_, N_2_O, N_2_ gases, or H_2_O, showing high selectivity to diamine against interfering gases or water, standing out as a promising material for environmental applications in electronic, optical or work function sensors for cadaverine detection, even in humid environments.

## Introduction

Cadaverine (Cad), discovered in 1885 by Ludwing Brieger, is a low-molecular-weight aliphatic diamine with five carbon atoms and two terminal amine groups (−NH_2_). Formed by the decarboxylation of the amino acid lysine by the enzyme lysine decarboxylase.^1^ Cad is a biogenic amine (BA) associated with the odors of dead bodies and spoiled food. In the human body, BAs are present in low concentrations in metabolic pathways and exert several essential physiological functions.^2,3^ However, high level of cadaverine can be marked as microbial spoilage products and can lead to many types of health risks such as vomiting, itching, skin irritation, headache, flushing, hypotension, hypertension, tachycardia, impaired breathing, asthma, and cancer.^4,5^ In the literature, Cad is noted as a biomarker of food freshness and environmental contamination.

As a biomarker, cadaverine helps ensure food and environmental safety by indicating food freshness and the presence of contamination. Although less toxic than other biogenic amines, such as histamine and tyramine, their ingestion can cause severe symptoms to the human body. Cytotoxic concentrations in food can cause intestinal cell necrosis. Interaction with dietary nitrites can form carcinogens and increase the toxicity of other amines, such as histamine, by inhibiting the detoxifying enzymes. Recent work on about the toxicity in vitro of the cadaverine to intestinal cells^6^ has shown that the concentrations often achieved in cheese, sausages, fish, and fish derivatives are cytotoxic (causing necrosis). It was concluded that food concentrations higher than 510 mg kg^–1^ may have harmful effects. Therefore, monitoring Cad levels in food and other media is crucial to maintaining public health and environmental safety.

There are several methods for monitoring the concentration of BAs in foods, including techniques such as thin-layer chromatography,^7,8^ capillary electrophoresis,^9^ electrochemical sensor,^10^ electrochemical biosensor,^11,12^ such as chromatography,^13^ HPLC (high-performance liquid chromatography) using coupled fluorescence detector,^14^ and potentiometric sensors.^15,16^ However, the above techniques require expensive instruments, enzymes, and high-cost electrodes. On the other hand, experimental studies have shown that nanocages such as B_12_N_12_ are possible and stable structures, and can be obtained from boron nitrides by top-down methods, such as plasma electrical discharge or plasma-assisted chemical vapor deposition.^17^ These techniques allowed nanocages to be obtained with high purity and morphology control. In addition, Oku and collaborators experimentally obtained BN nanocages modified with transition metals such as Fe, Y, Ag, and La.^18−20^ The literature shows that nanocages can be synthesized in the laboratory or even at scale and show promising characteristics for application in sensing. Furthermore, the reported synthesis uses known methods, and the nanocages require low-cost precursors (absence of noble metals) and can also be supported in small quantities by simple matrices such as graphene^21^ to form sensors, enabling their real application. The development of boron nitride materials, such as nanotubes (BNNT), two-dimensional (h-BN),^22,23^ nanoglomerates (B_*x*_N_*y*_),^24−26^ nanocage (B_12_N_12_),^26^ and their X_12_Y_12_ variants,^27−29^ as sensors for toxic gases and organic molecules, have attracted intense interest from the scientific community in recent years, due to their high sensitivity, rapid detection capability, and low recovery time.

X_12_Y_12_ nanocages have unique and differentiated properties, making them versatile nanomaterials for various applications, including catalysis, energy storage, and sensors.^30−32^ Furthermore, many works have been dedicated to the study and characterization of these nanocages modified with transition metals (TMs)^33−35^ to improve or generate new properties and thus expand their applications. Nanocages modified with Cu, for example, have been explored in several areas, including heterogeneous catalysis, energy storage, sensors, biosensors and even in biomedical applications, due to the properties acquired by the presence of the metal in the structure.^36−38^

Research published in recent years shows that interest in the development of new technologies for detecting BAs remains high, as can be seen in the work of Sudalaimani and collaborators,^39^ who proposed the colorimetric sensing of putrescin (Put) and Cad, using ninhydrin as a reagent for detecting food spoilage. Or in the research by Kim et al.,^40^ who monitored BAs in food spoilage using a graphene electronic matrix conjugated to a wireless chemical receptor. According to the authors, their matrix presents better sensitivity to Put and Cad than do commercial devices. More recently, an activated fluorescent sensor for detecting Put in fish samples using a thiazole derivative proposed by Lavanye and colleagues.^41^ The authors used a thiazole derivative to interact with putrescine and analyzed its photophysical properties by using UV and fluorescence spectroscopy. The electronic properties of the interaction are also studied with the aid of theoretical calculations at the DFT level.

In turn, recent theoretical work has studied the application of pure and Cu-modified X_12_Y_12_ nanocages for the detection of BAs, as found in the study by Silva et al., in which they analyzed the interactions between B_12_N_12_, B_12_P_12_, Al_12_N_12_, and Al_12_P_12_ nanocages with putrescine^42^ and the use of B_12_N_12_ nanocages decorated with Cu for detection of putrescine.^43^ Ferreira and collaborators^44^ developed a theoretical study on the detection of Cad and Put on the surface of carbon nanotubes (CNTs), boron and nitrogen nanotubes (BNNTs), and gallium and nitrogen nanotubes (GaNNTs); they indicated that CNT and BNNT systems can selectively detect Cad and Put in nature. However, despite the relevance of the topic and advanced studies with nanocages, no research has been found in the literature on the application of TM-modified X_12_Y_12_ nanocages for Cad detection. Therefore, based on the large and diverse application of B_12_N_12_ and Al_12_N_12_ nanocages reported in the literature,^28,30,33^ their potential to detect organic molecules and their promising response to putrescine, this work makes use of advanced density functional theory (DFT) level calculations to study the interaction between cadaverine and pure and Cu-modified Al_12_N_12_ and B_12_N_12_ nanocages, exploring the potential of nanocages for applications in chemoresistive, optical, and work function sensors.

## COMPUTATIONAL METHODOLOGY

Calculations at the DFT-D3^45^ level with the B3LYP hybrid functional and the 6-31G(d,p) base set were used for all systems to investigate the adsorption of the cadaverine molecule on the surfaces of the B_12_N_12_ and Al_12_N_12_ nanocages. The calculations were carried out for isolated nanocages and then for nanocages modified with copper in five different configurations,^34,38^ generating ten modified structures, which are

(I) Doped: with copper replacing Al or B atoms and also replacing an N atom (CuAl_11_N_12_, Al_12_N_11_Cu, CuB_11_N_12_, and B_12_N_11_Cu).
(II) Decorated: with the insertion of a copper atom in the external region of the nanocages, positioned on one of the bonds between the tetragonal and hexagonal rings (Cu(b_64_)Al_12_N_12_ and Cu(b_64_)B_12_N_12_) or on one of the bonds between two rings hexagonal (Cu(b_66_)Al_12_N_12_ and Cu(b_66_)B_12_N_12_).
(III) Encapsulated: with a copper atom positioned inside the nanocages (Cu@Al_12_N_12_ and Cu@B_12_N_12_).

It is noteworthy that the hybrid functional B3LYP, based on generalized gradient approximation (GGA) with 20% Hartree–Fock (HF), and the basis function set 6-31g(d,p) employed here are robust and widely used for the study of nanocages and suitable to describe the structures investigated in this work. They have been reported in recent studies with pure and Cu-modified B_12_N_12_ and Al_12_N_12_^28,30,35−38,43,58^ and in theoretical and hybrid studies of diamine adsorption with putrescine and cadaverine.^39,41−43^

The calculations were developed in the ORCA 5.0 package^46^ and the structure optimizations used RMS gradient, RMS displacement, maximum gradient and maximum displacement criteria: 5 × 10^–6^ hartree, 1 × 10^–4^ hartree/bohr, 2 × 10^–3^ bohr, 3 × 10^–4^ hartree/bohr, and 4 × 10^–3^ bohr. In addition, the vibrational frequencies were also calculated to guarantee the absence of imaginary frequencies and ensure that the optimized structures are ground states.

The affinity between the nanocages and Cu is investigated by the cohesion energy (*E*_coh_), which is calculated as eq 1:^47^
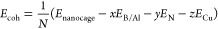
1where *E*_nanocage_ is the total energy of pure or Cu-modified nanocages; *E*_B/Al_, *E*_N_, and *E*_Cu_ are the energies of the B or Al, N and Cu atoms, respectively; *x*, *y* and *z* are the quantities of each atom (B or Al, N, and Cu, respectively) in the structures, and *N* is the total number of atoms.

The electronic sensitivity (Δ*E*_gap_) for the adsorption between Cad and the nanocages was calculated based on the highest occupied molecular orbital–lowest unoccupied molecular orbital (HOMO–LUMO) gap of the systems before and after adsorption, as follows (eq 2):
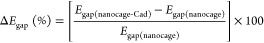
2where *E*_gap(nanocage-Cad)_is the energy gap of the nanocage-Cad, and *E*_gap(nanocage)_ is the gap of the pure or the modified nanocage. To calculate quantum mechanical descriptors such as chemical potential (μ), global hardness (η), and electrophilicity (ω), eqs 3–5^48−50^ were used:
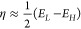
3
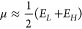
4
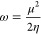
5

The stability study of the systems uses electrophilicity for the isolated and modified nanocages and also the free energy analysis of Gibbs (Δ*G*_ads_) for the systems after Cad adsorption, which is calculated according to eq 6:
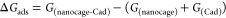
6where *G*_(nanocage-Cad)_ is the free energy after adsorption of cadaverine, *G*_(nanocage)_ is the free energy of the isolated cages, and *G*_(Cad)_ is the Gibbs free energy of cadaverine.

To investigate the potential application of nanocages as a material for chemiresistive and work function sensors for Cad detection, some specific parameters were calculated, such as the electrical conductivity (σ) of the nanomaterial, which is related to electronic sensitivity Δ*E*_gap_, work function (Φ), the Cad adsorption energy on the nanocages (*E*_ads_), the sensor recovery time (τ), and the sensitivity (*S*).

The electrical conductivity (σ) of the pure and modified nanocages before and after the adsorption of Cad molecules was calculated according to eq 7:^51^
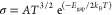
7where *A* (electron/m^3^K^3/2^) is a constant, *T* is the thermodynamic temperature (K), *E*_gap_ is the energy gap, and*k*_B_ is the Boltzmann constant (8.62 × 10^–5^ eV K^–1^). In turn, the variation in the electrical current density in the sensor (*j*) can be experimentally associated with the value of the work function (Φ) before and after the interaction of the nanocage with the molecule to be detected, according to eq 8:^34,52^
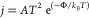
8where *A* is the Richardson–Dushman constant (theoretical value = 120.1 A m^–2^ K^–2^)^53^ and others have already been defined previously. The work function here is determined in terms of the frontier orbitals as in eq 9:
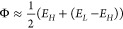
9

The adsorption energy (*E*_ads_) was calculated using eq 10:
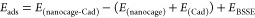
10where *E*_(nanocage-Cad)_is the energy of Cad adsorbed on Al_12_N_12_ or B_12_N_12_ isolated or modified with Cu, *E*_(nanocage)_ is the energy of the nanocage, *E*_(Cad)_ is the energy of cadaverine, and *E*_BSSE_ is the base overlap error energy (BSSE).

Using Cad sensitivity and adsorption results, the recovery time (τ) of the best system was determined, which is exponentially related to the *E*_ads_ value of the system and can be calculated using eq 11:^54,55^
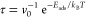
11where *v*_0_ is the attempt frequency (5.2 × 10^14^*v*_0_s^–1^)^42^ and the other parameters have already been defined previously.

In order to thoroughly evaluate the stability of nanocage–Cad interactions, the two systems that showed greater sensitivity to Cad were subjected to quantum molecular dynamics (MD) of 1000 ps, at room temperature (temperature = 298.15 K), with an integration interval of 2 fs, dump = 25.0, step = 1.0, nvt = true and shake = 2 (see Supporting Information), with an integration interval of 2 fs, in which the calculation of the force uses the GFN-1 Hamiltonian implemented in the xTB software.^56^ After analyzing the stability of the adsorption systems, the partial density of states (PDOS) and frontier molecular orbital (HOMO and LUMO) spectra were plotted for the best Cad adsorption system, using the MultiWfn package,^57^ to improve our understanding of the electronic characteristics of interaction.

With the aim of also evaluating the applicability of the system with the best result found and of pure B_12_N_12_ as a material for an optical Cad detection sensor, the time-dependent density functional theory method (TD-DFT) is used with 100 roots for the analysis of transition states and calculation of the UV–vis spectra of the optimized structures, in vacuum, before and after adsorption.

Another factor to be considered when designating a potential material for application in electrical or work function sensors is its selectivity. And to investigate the selectivity of the system for Cad adsorption against other gases and in the presence of humidity. The system that presented the best result for detecting Cad was also subjected to interaction with water and other gases considered interfering (NO, H_2_, H_2_S, CO, COCl_2_, N_2_O and N_2_). Selectivity was evaluated by calculating the response of sensor (*S*) and the selectivity coefficient (κ), using eqs 12 and 13, as follows:
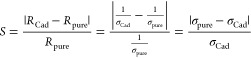
12
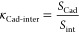
13where σ_Cad_ represents the conductivity of the nanocage/Cad system, σ_pure_ is the conductivity of the isolated nanocage, and *R* is the resistance, which is inversely proportional to the electrical conductivity, which is defined in eq 7.

The selectivity for the work function (*S*_*j*_) was also calculated, for the adsorption of Cad and interferents, as a function of the electrical current density in the sensor (*j*), according to eq 14 below, as well as the selectivity coefficient (κ_*j*_) for this parameter, with based on eq 13.
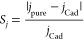
14

## Results and Discussion

### Structural Analysis of Pure and Cu-Modified Nanocages

**Figure 1:**
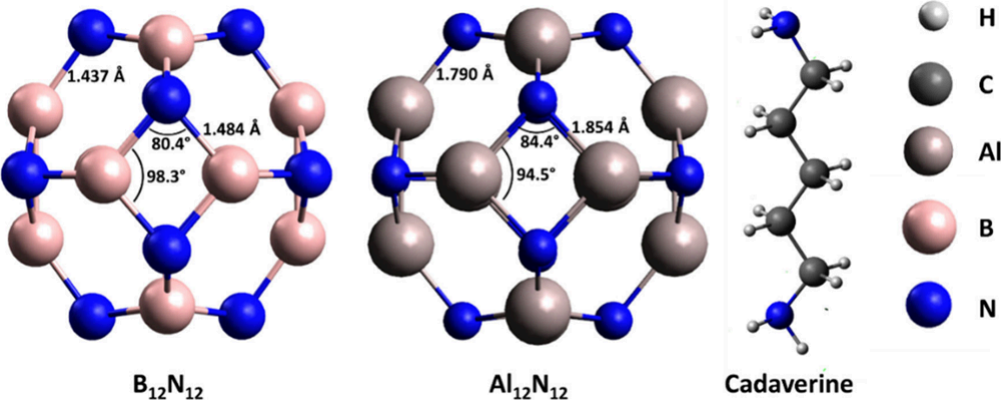
Structures of the optimized B_12_N_12_, Al_12_N_12_, and cadaverine nanocages.

Initially, the B_12_N_12_ and Al_12_N_12_ nanocages were optimized at the DFT level, as well as the cadaverine (Cad) molecule, as shown in Figure 1. The nanocages are formed by six tetragonal rings and eight hexagonal rings each. In the B_12_N_12_ nanocage, the internal angles calculated for the tetragonal rings measure B–N–B = 80.4° and N–B–N = 98.3°. In the Al_12_N_12_ nanocage, the calculated internal angles of the four-membered rings measure Al–N–Al = 84.4° and N–Al–N = 94.3°. Thus, in the nanocages there are two distinct types of bonds: located between a hexagonal ring and a tetragonal ring (b_64_) and between two hexagonal rings (b_66_), which measure respectively: 1.484 and 1.437 Å in the B_12_N_12_ cage and 1.854 Å and 1,790 Å in Al_12_N_12_. The cadaverine molecule is a diamine with a four-carbon chain.

The most stable optimized structures of B_12_N_12_ and Al_12_N_12_ nanocages present high symmetry and a dipole moment (DM) calculated equal to zero 0.00 and 0.02 D, respectively. The bond distances *d*_(B–N)_ calculated for B_12_N_12_ and d_(Al–N)_ for Al_12_N_12_ are in agreement with the values found by Rad and collaborators^28^ in their theoretical research at the DFT level and other more recent works.^58−62^ as well as being comparable to the experimental results obtained by mass spectroscopy.^17^ The calculated angles BNB and NBN also agree with data presented in the literature.^60,61^ The Cad molecule, after geometry optimization, has an electric dipole (DM = 1.68 D) and a HOMO–LUMO gap (*E*_gap_ = 8.41 eV).

**Figure 2:**
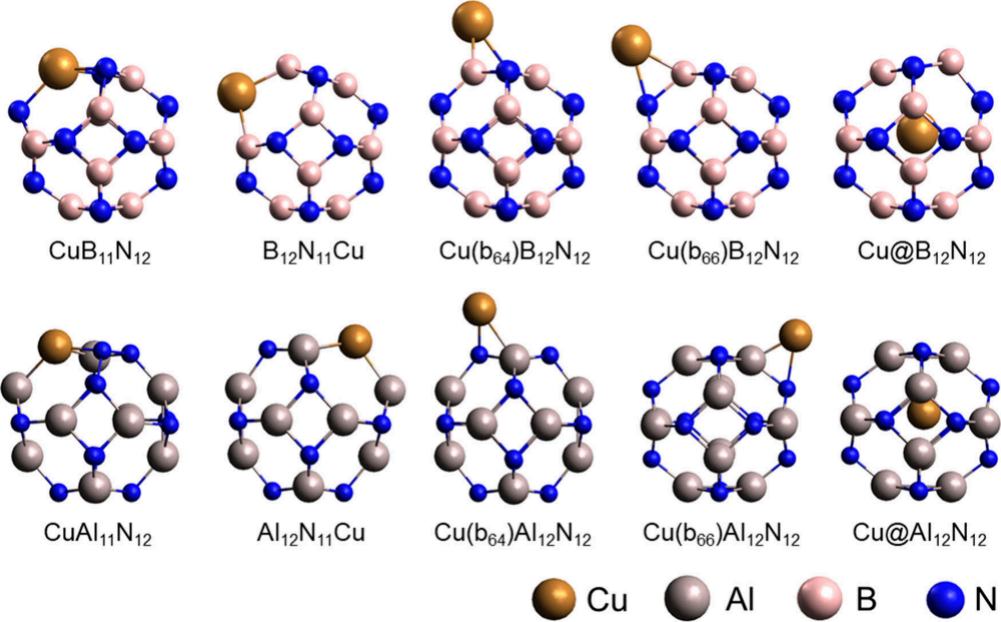
Optimized structures of Al_12_N_12_ and B_12_N_12_ nanocages modified with Cu.

With the interest of investigating the influence of a metallic atom on the structure and its ability to improve the electronic properties of the nanocages, their strategic modification with the copper occurred in five different positions in each nanocage in order to generate 10 modified structures (CuB_11_N_12_, B_12_N_11_Cu, CuAl_11_N_12_, Cu(b_64_)B_12_N_12_, Cu(b_66_)B_12_N_12_, Cu@B_12_N_12_, Al_12_N_11_Cu, Cu(b_64_)Al_12_N_12_, Cu(b_66_)Al_12_N_12_ and Cu@Al_12_N_12_), as shown in Figure 2.

**Figure 3:**
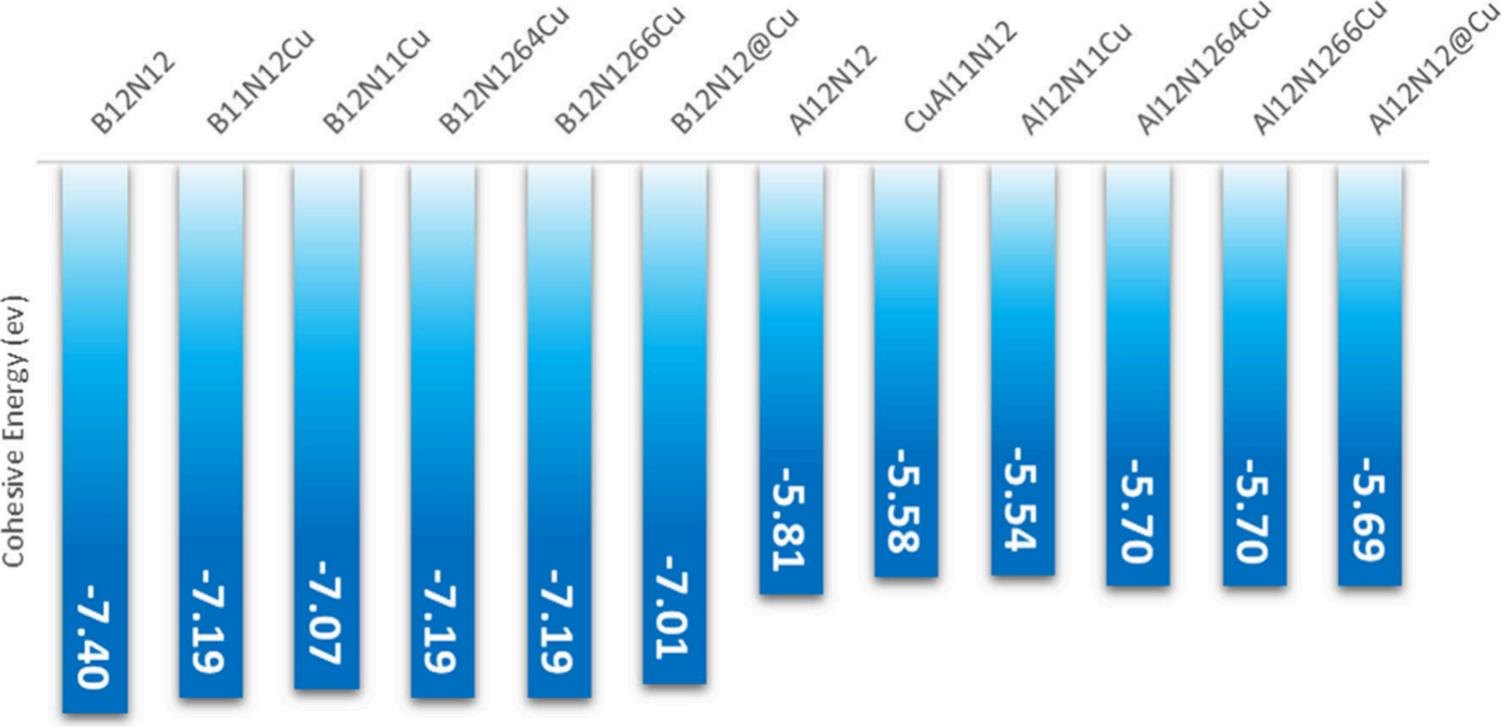
Cohesion energy (*E*_coh_) of Cu-modified Al_12_N_12_ and Cu-modified B_12_N_12_ nanocages.

The nanocages modified with copper showed some deformations in their structure after geometry relaxation; this effect is more prominent in systems doped with copper instead of N, B_12_N_11_Cu and Al_12_N_11_Cu, where the metal projects out of the cages. In decorated nanocages, changes in bond lengths and deformation of rings occur adjacent to the interaction with the metal. While in encapsulated systems, the metal moved from the center of mass during geometry relaxation.^38^ The cohesion energy (*E*_coh_) for the Cu-modified systems was calculated to investigate the degree of interaction of the metal with the nanocages. The results are plotted in Figure 3. It is clearly observed that the metal interacts better with the B_12_N_12_ nanocage when compared to the interactions with Al_12_N_12_. It is also noteworthy that the cohesion energies of the isolated nanocages, B_12_N_12_ and Al_12_N_12_, present higher values than those of the modified cages, −7.40 and −5.81 eV, respectively. Which indicates that the modifications make the nanocages more reactive. And this characteristic has been commonly observed in the literature.^33−35,43,58,60^ It is worth noting that in modified systems, when copper is positioned outside the cage, it interacts more strongly with the cage. In other words, systems with decorated copper have greater cohesion and, therefore, greater stability among the modified nanocages.

### Stability Analysis of Nanocages

The theoretical prediction of B_12_N_12_ was performed by Jensen and Toftlund, who showed that the most stable cluster consisted of eight hexagonal rings and six tetragonal rings,^63^ as used here. B_12_N_12_ nanocages were first synthesized by Golberg et al.^64^ and isolated by Oku and co-workers.^17^ In addition, other syntheses and modifications of fullerene-like nanocages are reported in the literature,^30,32,65^ including experimental applications, such as hydrogen storage.^66,67^ Moreover, Zhu et al.^65^ reported the large-scale production of BN nanocages, using a B–N–O precursor. Given the experimentally proven stability of the nanocages and their differentiated characteristics, the modification of nanocages with metals such as copper is a strategy explored in several works reported in the literature for various applications.^35,38,43,62^

Some theoretical works have investigated the modification of B_12_N_12_ and Al_12_N_12_ with all the metals of the first transition row;^36,58,62^ these use parameters such as binding energy (*E*_b_), cohesion energy (*E*_coh_), quantum descriptors (η, μ, ω), and molecular dynamics (MD), to show that the modified nanocages are stable. However, we have shown in previous works, using MD, that the nanocages with encapsulated Cu are the least stable of the series,^34^ corroborating with data from other works^68^ and that the configuration (b_64_) is more stable than (b_66_),^34,43^ as also observed by Arshad.^58^ Similarly, quantum descriptors such as hardness (η), chemical potential (μ), and electrophilicity (ω) were calculated here, to analyze the stability of the modified systems, as applied in recent works available in the literature,.^35,68,69^ In addition to their dipole moment (DM), the charges acquired by the Cu atoms were also calculated according to the Loewdin scheme in each nanocage. The results are shown in Table 1.

**Table 1:** Chemical Hardness (η), Chemical Potential (μ), Electrophilicity (ω), Dipole Moment (DM), and Charge on Cu (*Q*_Cu_) of the Modified Al_12_N_12_ and B_12_N_12_ Nanocages

system	η (eV)	μ (eV)	ω (eV)	DM (Debye)	*Q*_Cu_
B_12_N_12_	3.43	–4.19	2.56	0.00	–
CuB_11_N_12_	1.02	–5.86	16.78	1.26	0.37
B_12_N_11_Cu	1.38	–3.78	5.16	2.07	0.02
Cu(b_64_)B_12_N_12_	1.52	–3.25	3.48	1.43	0.14
Cu(b_66_)B_12_N_12_	1.56	–3.18	3.25	1.67	0.14
B_12_N_12_@Cu	2.78	–4.13	3.06	2.88	0.04
Al_12_N_12_	2.00	–4.42	4.90	0.02	–
CuAl_11_N_12_	0.95	–3.51	5.84	0.80	0.20
Al_12_N_11_Cu	1.01	–3.91	7.55	2.65	–0.34
Cu(b_64_)Al_12_N_12_	1.17	–3.45	5.07	1.52	0.06
Cu(b_66_)Al_12_N_12_	1.11	–3.42	5.29	2.31	0.05
Al_12_N_12_@Cu	1.57	–4.69	6.99	1.67	0.58

The calculated chemical hardness values reveal that B_12_N_12_ (η = 3.43 eV) is a harder species than all other cages, including the Al_12_N_12_ nanocage (η = 2.00 eV). The modified nanocages with the highest and lowest hardness are B_12_N_12_@Cu and CuAl_11_N_12_, (η = 2.78 and 0.95 eV, respectively). When analyzing the values of the chemical potential (μ) there is an inversion, when the Al_12_N_12_ nanocage presents a higher potential (more negative) than the pristine B_12_N_12_ and the other systems.

Based on the principles of maximum hardness (η)^49^ and minimum electrophilicity (ω),^50^ according to Parr and Person,^69^ systems with greater chemical hardness and lower electrophilicity are more stable. Thus, electrophilicity is a parameter for analyzing the reactivity or stability. After interaction with copper, the modified nanocages showed higher electrophilicity values compared with the pure cages. This new parameter confirms the inference made from the *E*_coh_ analysis, which indicated greater reactivity of the modified nanocages. Finally, the CuB_11_N_12_ nanocage appears to be the most reactive of the series, as shown in Table 2. This high reactivity is justified by its low hardness (η = 1.02 eV) and high (more negative) chemical potential (μ = −5.86 eV). The same system also appears to be more reactive when B_12_N_12_ is modified with all 10 metals from the first transition row.^38^

**Table 2:** HOMO (*E*_H_), LUMO (*E*_L_), Energy Gap (*E*_gap_), Gap Variation (Δ*E*_gap_) and Electronic Sensitivity (Δgap) of the Systems before and after Cadaverine Adsorption

		Isolated			Cad Adsorbed				
system	*S*	*E*_H_ (eV)	*E*_L_ (eV)	*E*_gap_ (eV)	*E*_H_ (eV)	*E*_L_ (eV)	*E*_gap_ (eV)	Δ*E*_gap_ (eV)	Δgap (%)
B_12_N_12_	α	–7.63	–0.76	6.87	–6.57	0.15	6.72	0.15	2.18
CuB_11_N_12_	α	–6.89	–4.84	2.05	–6.34	–3.63	2.70	0.65	31.71
B_12_N_11_Cu	α	–5.16	–2.40	2.76	–4.39	–1.59	2.80	0.04	1.45
Cu(b_64_)B_12_N_12_	α	–4.78	–1.73	3.04	–4.58	–0.34	4.25	1.21	39.80
Cu(b_66_)B_12_N_12_	α	–4.74	–1.62	3.12	–4.08	–0.22	3.86	0.74	23.72
B_12_N_12_@Cu	α	–6.91	–1.34	5.57	–6.14	–0.46	5.68	0.11	1.97
Al_12_N_12_	α	–6.42	–2.43	3.99	–5.89	–1.93	3.97	0.02	0.50
CuAl_11_N_12_	α	–4.45	–2.56	1.89	–3.97	–2.00	1.96	0.07	3.70
Al_12_N_11_Cu	α	–4.92	–2.90	2.02	–4.42	–2.10	2.32	0.30	14.85
Cu(b_64_)Al_12_N_12_	α	–4.62	–2.28	2.35	–3.37	–1.79	1.58	0.77	32.77
Cu(b_66_)Al_12_N_12_	α	–4.53	–2.32	2.22	–3.27	–1.84	1.43	0.79	35.59
Al_12_N_12_@Cu	β	–6.27	–3.12	3.15	–5.58	–2.53	3.05	0.10	3.17

The dipole moment values calculated for the modified systems are consistent with the geometry changes observed in the optimization stage, where there are greater values of DM for the B_12_N_11_Cu and Cu@B_12_N_12_ systems, indicating a lower charge distribution in the nanocage. In the modified Al_12_N_12_ systems, the CuAl_11_N_12_ and Cu(b_66_)Al_12_N_12_ nanocages have higher DM. The charges acquired by the Cu atoms in the nanocages present positive values, indicating that electronic transfer occurs from the metal to the cage. This trend was also observed by Arshad et al.,^58^ when studying B_12_N_12_ decorated with transition metals. In contrast, in the Al_12_N_11_Cu system, the charge is negative and moves in the opposite direction (*Q*_Cu_ = −0.34), that is, from the cage to the metal. This happens because aluminum is less electronegative than copper, which, in the Al_12_N_11_Cu nanocage, removes electrons from the three adjacent Al atoms.

### Cadaverine Adsorption: Electronic Sensitivity, Work Function, and Adsorption Energy

**Figure 4:**
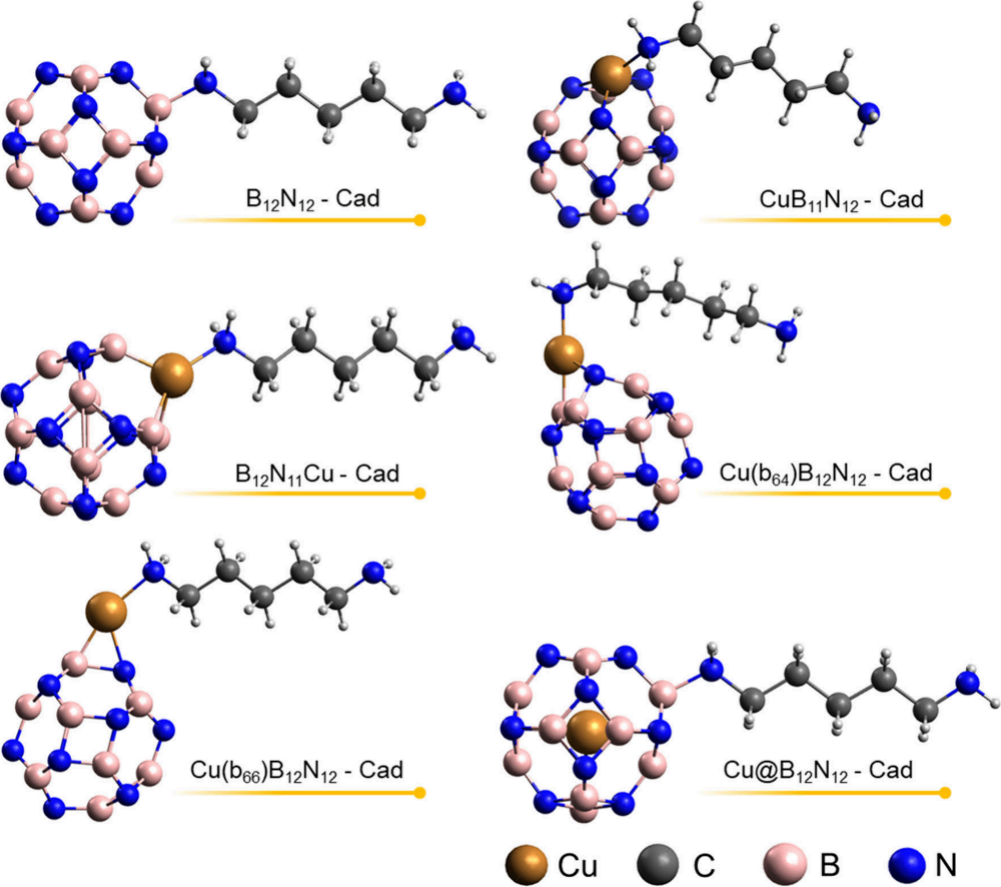
Optimized structures of Cad adsorption on pure and Cu-modified B_12_N_12_.

**Figure 5:**
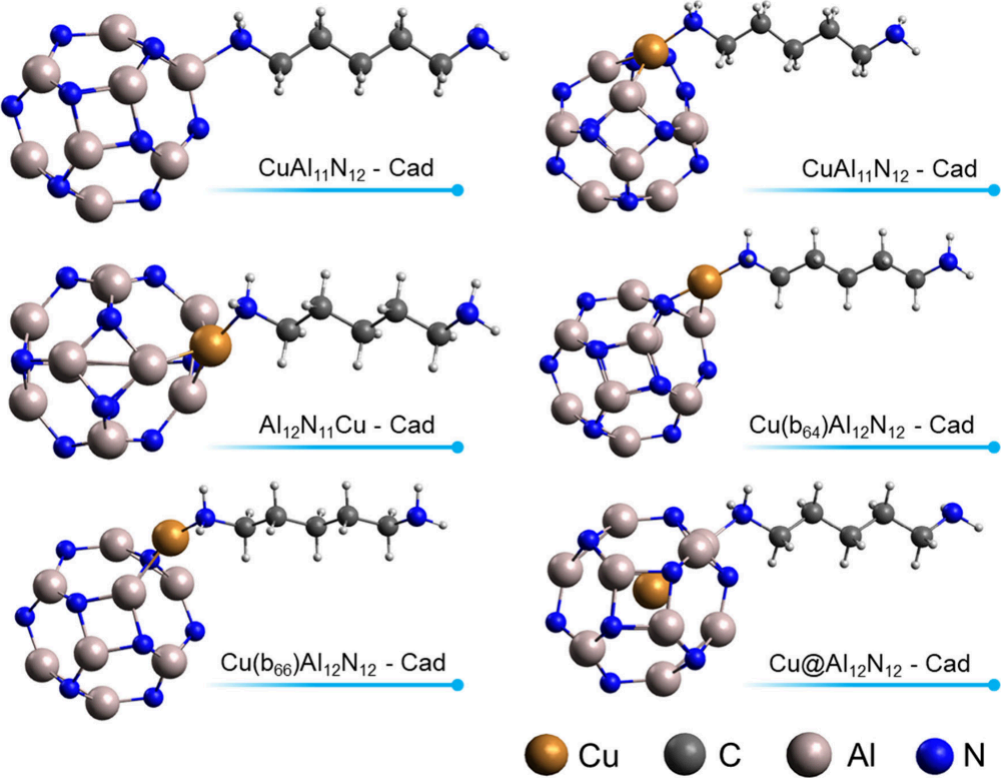
Optimized structures of Cad adsorption on pure and Cu-modified Al_12_N_12_ nanocages.

After the modified nanocages and the Cad molecule were optimized, the diamine was adsorbed on the surface of the nanocages, as can be seen in the images of the optimized structures in Figures 4 and 5. In Figure 4, it is observed that Cad binds to Cu atoms in the doped and decorated structures, binding to B atoms in the pure B_12_N_12_ and Cu@B_12_N_12_ nanocages. The adsorption preference of Cad over Cu and B atoms is justified by the fact that these are more electrophilic sites than nitrogen sites. In Figure 5, we have the adsorption of Cad on pure and modified Al_12_N_12_ nanocages. Diamine binds through one of its amino groups to the Cu and Al sites of the cages. It is observed that for both BN and AlN systems, during adsorption, the nitrogen Cad amino group makes four bonds, which suggests that the free electron pair of nitrogen is used in the interaction.

The energies of the HOMO and LUMO frontier orbitals of the systems were collected to determine the energy gap values (*E*_gap_) of each one and the gap variation (Δ_ap_), before and after Cad adsorption, as well as the variation of the gap in percentage (Δgap (%)), which can be related to the sensitivity of the system to Cad adsorption. Electrical conductivity (σ) is a significant parameter to be considered in gas chemiresistive sensor applications and can be experimentally calculated with based on eq 7. The obtained values of *E*_L_, *E*_H_, *E*_gap_, Δ*E*_gap_, and Δgap, are summarized in Table 3. It is clearly observed that the energies of the HOMO and LUMO orbitals were changed after the interaction with Cad, and this change directly reflected in the variation of *E*_gap_. B_12_N_12_, Cu(b_66_)B_12_N_12_, Cu(b_64_)Al_12_N_12_, Cu(b_66_)Al_12_N_12_), and encapsulated (Cu@B_12_N_12_ and Cu@Al_12_N_12_). The *E*_gap_ values for the spin-doublet refer to molecular orbitals (spin-up α or spin-down β) of the system with greater electronic sensitivity (Δgap).^38^

**Table 3:** Work Function before (Φ) and after Adsorption (Φ_ads_), Variation of the Work Function (ΔΦ) of the Systems, Charges of Cu (*Q*_Cu_) and Cad (*Q*_Cad_), and Variation of Gibbs Free Energy (Δ*G*_ads_) and Adsorption Energy (*E*_ads_)

system	Φ (eV)	Φ_ads_ (eV)	ΔΦ (%)	*Q*_Cu_	*Q*_Cad_	Δ*G*_ads_ (eV)	*E*_ads_(eV)
B_12_N_12_–Cad	4.19	3.21	23.39			–1.01	–1.55
CuB_11_N_12_–Cad	5.86	4.98	15.02	+0.431	+0.293	–1.35	–1.67
B_12_N_11_Cu–Cad	3.78	2.99	20.90	–0.166	+0.237	–0.95	–1.18
Cu(b_64_)B_12_N_12_–Cad	3.25	2.46	24.31	+0.257	+0.2	–1.26	–1.39
Cu(b_66_)B_12_N_12_–Cad	3.18	2.15	32.39	+0.206	+0.198	–0.96	–1.02
Cu@B_12_N_12_–Cad	4.13	3.30	20.10	–0.306	+0.354	–1.30	–1.81
Al_12_N_12_–Cad	4.42	3.91	11.54			–1.25	–1.72
CuAl_11_N_12_–Cad	3.51	2.98	14.98	+0.279	+0.246	–1.12	–1.27
Al_12_N_11_Cu–Cad	3.91	3.26	16.62	–0.336	+0.289	–1.07	–1.16
Cu(b_64_)Al_12_N_12_–Cad	3.45	2.58	25.22	+0.137	+0.237	–0.97	–1.07
Cu(b_66_)Al_12_N_12_–Cad	3.42	2.55	25.44	+0.146	+0.243	–0.95	–1
Cu@Al_12_N_12_–Cad	4.69	4.05	13.65	+0.156	+ 0.330	–1.40	–1.9

The results show that isolated B_12_N_12_ presents a gap energy (*E*_gap_ = 6.87 eV), while pure Al_12_N_12_ presents a gap energy (*E*_gap_ = 3.99 eV). A reduction in the *E*_gap_ value of the systems was observed after modification with copper. Therefore, the greatest reduction is recorded for the doped systems: CuB_11_N_12_ (4.82 eV) and B_12_N_11_Cu (4.11 eV), CuAl_11_N_12_ (2.10 eV) and Al_12_N_11_Cu (1.97 eV), followed by the decorated systems: Cu(b_64_)B_12_N_12_ (3.83 eV), Cu(b_66_)B_12_N_12_ (3.75 eV), Cu(b_64_)Al_12_N_12_ (1.64 eV), Cu(b_66_)Al_12_N_12_ (1.77 eV), and the smallest band-gap reductions were observed for nanocages with copper-encapsulated Cu@B_12_N_12_ (1.30 eV) and Cu@Al_12_N_12_ (0.84 eV). The gap reduction observed for the modified systems shows that (i) the nanocages are electronically influenced by the presence of copper in the structure and (ii) the metal makes them more reactive and increases their electrical conductivities.

Through the adsorption of the Cad molecule on the surface of the nanocages, a further reduction in the gap was observed for the systems B_12_N_12_–Cad, CuAl_11_N_12_–Cad, and Cu(b_66_)Al_12_N_12_–Cad, while the gap remains unchanged for Cu@Al_12_N_12_–Cad, indicating that it is not sensitive to the presence of Cad and increases for other systems. Changes in *E*_gap_ are often used to evaluate the potential of pure and modified nanocages as conductometric sensors, and the greater decrease in *E*_gap_ upon interaction is associated with an increase in electrical conductivity,^70^ as reported by Hussain et al.,^37^ Janjua et al.,^71^ Ammar et al.,^72^ Rad and Ayub,^73^ and Abdolahi et al.^74^ The calculated sensitivity of pristine B_12_N_12_ and Al_12_N_12_ was 2.18% and 0.5%, respectively. The low sensitivity values found are equivalent to data published in the literature^42^ for the adsorption of a similar diamine, putrescine, with a sensitivity of 8.49% for B_12_N_12_ and 3.14% for Al_12_N_12_.

Systems with greater sensitivity to the Cad molecule are Cu(b_64_)B_12_N_12_ (Δgap = 39.8%) and Cu(b_66_)Al_12_N_12_ (Δgap = 35.59%). The sensitivity results found in this study are consistent with results published in the literature. Ferreira et al.^44^ used carbon nanotubes (CNTs) and boron nitrogen nanotubes (BNNTs) for the detection of putrescine and cadaverine. They show that CNTs are not sensitive to Cad and that BNNT showed a sensitivity of 9.3%. In their theoretical work at the DFT level, Silva et al.^43^ studied copper-modified B_12_N_12_ nanocages for putrescine adsorption. The authors reported a calculated sensitivity of 24.26% using the B3LYP functional and 23.54% using the B3PW91 functional. The authors also investigated the use of Minnisota functionals with variation in the Hartree–Fock (HF) percentage, such as M06-L/6-31G(d,p), M06/6-31G(d,p), and M06-2XL/6-31G(d,p), but the sensitivity found was not greater than 26%. These data suggest that (i) the functional and base set used here are the most suitable for the systems studied and, furthermore, (ii) the results obtained here present an improvement in the sensitivity for detecting diamines as the cadaverine.

The PDOS graphs were plotted for the modified systems before and after Cad adsorption (see Figures S1 and S2 in the Supporting Information). They show the electronic behavior of the systems during the cage–Cad interaction process. It is possible to observe in Figure S1, the reduction of the energy gap after interaction with Cu and that the nanocage contributes more than the metal to the formation of the LUMO of the system, which suggests a greater electron density donation with the adsorption of Cad, with the charge transfer from the diamine to the modified nanocages (BN and AlN), as will be shown below. The metal presents a greater contribution to the formation of the HOMO, which is related to the charge donation observed from Cu to the nanocage. Note that, in the Cu(b_64_)B_12_N_12_ and Cu(b_66_)B_12_N_12_ nanocages, a higher contribution of the metal to the formation of the HOMO is observed, which can be related to the greater sensitivity to diamine shown in Table 3. Figure S2 shows the predominant contribution of the nanocage to the formation of the frontier orbitals of the systems, which reinforces the transfer of electron density from Cad to the nanocage.

Regarding the extrapolation of the theoretical results obtained here to experimental applications, note that experimental results for this process have not yet been found in the literature. However, it is important to note that the electronic sensitivity results are indicative of success in the proposal of materials as sensors, but there may be significant changes when they are tested experimentally. This is due to the fact that small changes in the crystal structure of the synthesized nanocages are enough to influence the electrical conduction behavior of the materials. In addition, the structural configuration of the materials can change their characteristics as sensors, such as whether the material is composed of nanoparticles or a nanometer-scale thin film.

The work function values, before (Φ) and after (Φ_ads_) the adsorption of cadaverine, as well as the variation in the work function (ΔΦ) of the systems, were calculated to investigate the potential application of nanocages as sensors. The charges on the metal (*Q*_Cu_) and on cadaverine (*Q*_Cad_) were collected after adsorption and are listed in Table 4. In pure and modified systems, the highest work function value was recorded for the B_11_N_12_Cu nanocage and the lowest value was recorded for Cu(b_66_)B_12_N_12_. Likewise, the same systems present higher and lower values of Φ after Cad adsorption (4.98 and 2.15 eV, respectively). And in relation to the work function variation, which is related to the variation in current density in the sensor, the largest variation was recorded for Cu(b_66_)B_12_N_12_–Cad (32.4%), followed by Cu(b_66_)Al_12_N_12_–Cad (25.4%) > Cu(b_64_)Al_12_N_12_–Cad (25.2) > Cu(b_64_)B_12_N_12_–Cad (24.3%) > B_12_N_12_–Cad (23.4%). Silva et al.^42^ reported similar values of work function variation in his work: for Al_12_N_12_, (ΔΦ = 19.5%) and for B_12_N_12_, (ΔΦ = 15.2%).

**Table 4:** Wavelength (λ_max_), Oscillator Strength (*f*), Energy (*E*) and Main Electronic Transitions Associated with the Absorption Peaks of the Systems B_12_N_12_, Cu(b_64_)B_12_N_12_ and Cu(b_64_)B_12_N_12_–Cad

system	λ_max_ (nm)	*f*	*E* (eV)	transition
B_12_N_12_	174.4	0.039	7.1	H(α) → L(α) (58%)
				H-1(α) → L + 1(α) (33%)
	198.1	0.16	6.3	H(α) → L(α) (39%)
				H-1(α) → L + 1(α) (35%)
				
Cu(b_64_)B_12_N_12_	212	0.012	5.8	H(β) → L(β) (28%)
				H(α) → L(α) (21%)
	250.8	0.27	4.8	H(α) → L(α) (94%)
	332.1	0.031	3.7	H(α) → L(α) (91%)
				
Cu(b_64_)B_12_N_12_–Cad	215.6	0.011	5.7	H(α) → L(α) (28%)
				H-1(α) → L + 1(α) (20%)
	350.0	0.12	3.5	H(α) → L(α) (90%)
	373.3	0.026	3.3	H(α) → L(α) (60%)
				H(β) → L(β) (34%)

In the analysis of variation in copper charge (before and after Cad adsorption), we observed that, in systems where copper replaces nitrogen in the cage and in encapsulated systems, copper gains electronic density, becoming negatively charged. Therefore, Cu@Al_12_N_12_–Cad is the system in which copper receives the most charge from Cad adsorption (Δ*Q* = −0.42). in the other nanocages, copper gives up even more charge to the system, so that the greatest charge transfer is observed for Cu(b_64_)B_12_N_12_ (ΔQ = +0.116), followed by Cu(b_66_)Al_12_N_12_ (Δ*Q* = +0.097), which are also the systems that showed greater sensitivity to Cad. Regarding the charge acquired by the cadaverine molecule after adsorption in the nanocages, it appears that the Cad molecule injects charge into the nanocage, acquiring a positive residual charge. And the greatest electrical charge transfer occurs when Cad interacts with nanocages with copper encapsulated Cu@B_12_N_12_–Cad (Δ*Q* = +0.35) and Cu@Al_12_N_12_–Cad (Δ*Q* = +0.33). In this way, it is observed that the nanocages function as a Lewis acid, receiving electrons, and Cad acts as a Lewis base, providing electron density to the cage.

Gibbs free energy (Δ*G*_ads_) was calculated to investigate the stability of the adsorption systems. The adsorption energy (*E*_ads_) was also calculated and provides information about the intensity of the Cad interaction with the surface of the modified nanocages. *E*_ads_ is presented here already corrected with the base overlap error (BSSE). The variation in the Gibbs free energy (Δ*G*_ads_, Table 4) after adsorption showed negative values, indicating that adsorptions are spontaneous processes and generate stable products. The Cu@Al_12_N_12_–Cad and B_11_N_12_Cu–Cad systems showed the highest (most negative) values in the series (Δ*G*_ads_ = −1.40 and −1.35 eV, respectively). The literature shows that adsorption energy values lower than −0.3 eV characterize relatively weak interactions, of the physisorption type.^75^ The calculated adsorption energy shows that cadaverine adsorbs strongly on the surface of the nanocages, interacting via chemisorption, corroborated by good charge transfer between the species.

### Molecular Dynamics and Stability of Systems

**Figure 6:**
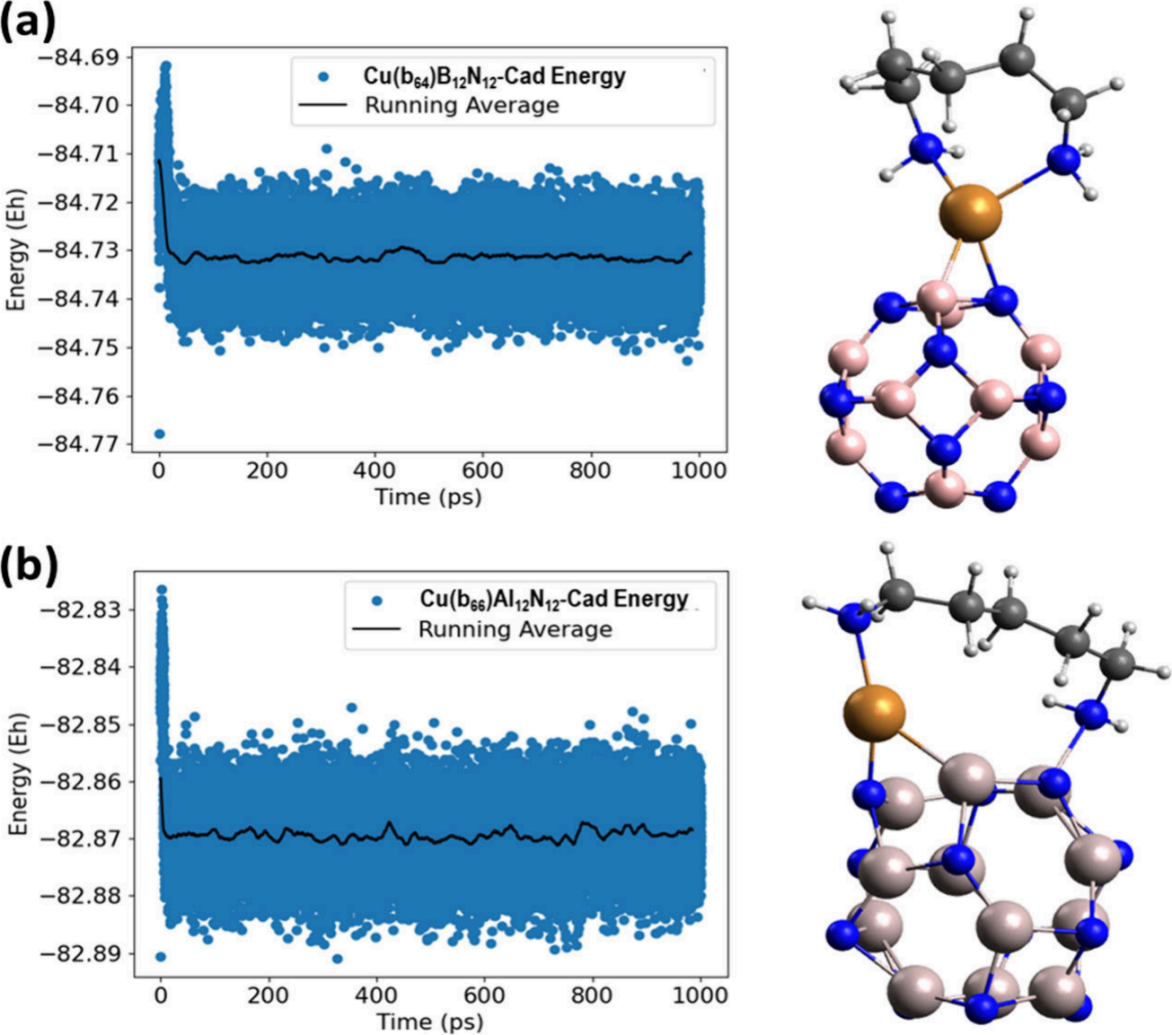
Quantum molecular dynamics of the Cu(b_64_)B_12_N_12_–Cad (a) and Cu(b_66_)Al_12_N_12_–Cad (b) adsorption systems.

Systems with greater sensitivity for adsorption of the cadaverine molecule were established: that is, Cu(b_64_)B_12_N_12_–Cad and Cu(b_64_)Al_12_N_12_–Cad. These were subjected to quantum molecular dynamics of 1000 ps, with an integration time of 2 ps, and the energy values of the systems at each step of the dynamics were plotted in Figure 6 (in blue) with the average variation observed for 200 intervals (in black), alongside the structural changes observed in the molecules. The dynamics of the Cu(b_64_)B_12_N_12_–Cad system is shown in Figure 6a, starting from the point with Cad linked to the copper of the nanocage through the N of one of its amino groups (−NH_2_). With less than 50 ps of quantum dynamics, it was observed that the Cad molecule establishes a second bond with the metallic center, through the nitrogen of the second amino group, remaining with its stable structure until the end of the dynamics is carried out. However, having gone through a few steps of the dynamics of the Cu(b_66_)Al_12_N_12_–Cad system (Figure 6b), the diamine binds to an Al atom neighboring the copper, also through the nitrogen of its other amino group. A configuration change is observed in the nanocage when the Cu atom leaves position b_66_ and moves to position b_64_, remaining there until the end of the dynamics. This conformational change from the b_66_ system to the b_64_ system is known and reported in the literature for Cu-decorated B_12_N_12_ nanocages.^35,38,43^ However, no reports of the change in Al_12_N_12_ systems were found. Another characteristic worth highlighting is related to the stabilization energy level of the systems. It is observed that the Cu(b_64_)B_12_N_12_–Cad system is more stable than the Cu(b_64_)Al_12_N_12_–Cad system (−84.8 and −82.8 Eh, respectively). In short, the Cu(b_66_)Al_12_N_12_ system is not stable, converting into b_64_ during molecular dynamics, which has lower sensitivity (32.77%), not being the most suitable for Cad adsorption and cyclic sensing. Meanwhile, the Cu(b_64_)B_12_N_12_ system proved to be stable and suitable for the adsorption of diamine.

### PDOS Analysis and Recovery Time

**Figure 7:**
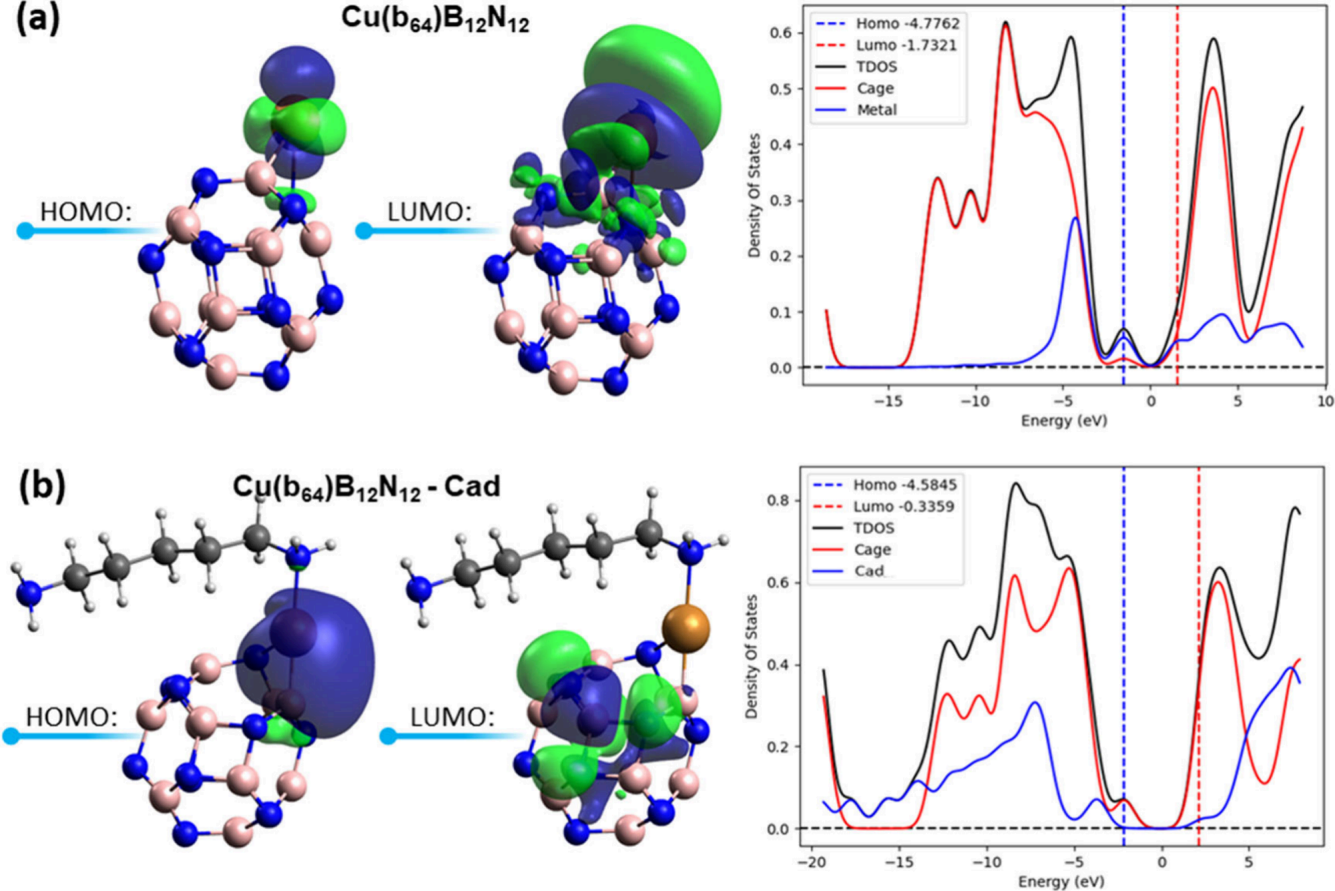
HOMO, LUMO, and PDOS of the Cu(b_64_)B_12_N_12_ nanocage before and after Cad adsorption.

In Figure 7, we have the HOMO and LUMO molecular orbitals, associated with the PDOS graphs of the Cu(b_64_)B_12_N_12_ nanocage, before and after Cad adsorption. The PDOS was plotted using the Multwfn software. It is observed that the HOMO molecular orbital of the isolated nanocage is predominantly located over the metallic center, while the LUMO orbital is distributed between the cage and the Cu. The contribution to the formation of frontier orbitals can be confirmed in the PDOS diagram, where a greater contribution of the cage to the formation of HOMO and an equivalent contribution of the cage and metal to the formation of LUMO of the isolated Cu(b_64_)B_12_N_12_ nanocage are observed. After interaction with the Cad molecule, there is a predominant contribution of the cage to the formation of HOMO and mainly LUMO. This may be related to the charge transfers from Cad and copper to the nanocage already reported, as well as the cage’s performance as a Lewis acid.

**Figure 8:**
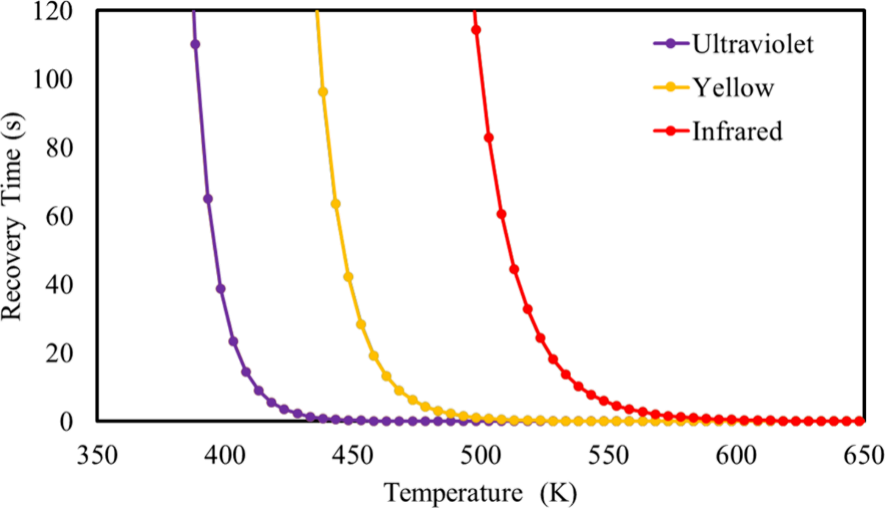
Variation in recovery time of the Cu(b_64_)B_12_N_12_–Cad adsorption system, as a function of the temperature and attempt frequency.

The recovery time of an adsorption system is directly influenced by the adsorption energy, temperature, and the attempt frequency used. Under these conditions, Figure 8 shows the variation in the recovery time of the Cu(b_64_)B_12_N_12_–Cad system (in seconds), given a temperature variation from 350 K to 650 K, considering the frequencies in the ultraviolet region, yellow and infrared.

The recovery time graph (τ) of the Cu(b_64_)B_12_N_12_–Cad system shows that, at ∼550 K, Cad spontaneously adsorbs from the nanocage within 5 s under infrared radiation. Under yellow radiation, τ tends to 0 at 500 K. However, in ultraviolet radiation, the recovery time is <1 s at 440 K. On the other hand, at room temperature and yellow light, the time for cadaverine desorption is greater than 12 h. which makes it impossible to apply the Cu(b_64_)B_12_N_12_ nanocage as a sensor for permanent use in the detection of cadaverine, since the adsorption of diamine on the surface of the nanocage inactivates it for a prolonged time. However, the Cu(b_64_)B_12_N_12_ nanocage is stable and suitable for strategic application in disposable sensors for rapid cadaverine detection, taking advantage of its high sensitivity, good adsorption energy, and high stability.

### UV–vis Adsorption Spectrum Analysis

**Figure 9:**
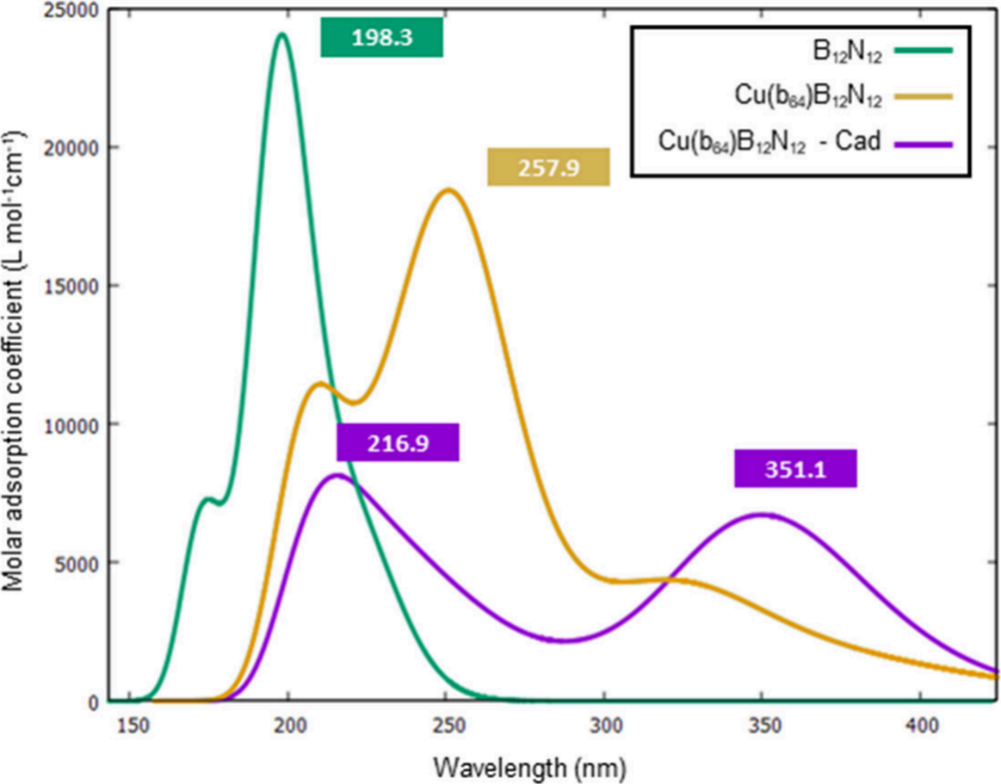
UV–vis absorption spectrum of the systems B_12_N_12_, Cu(b_64_)B_12_N_12_, and Cu(b_64_)B_12_N_12_–Cad.

Another potential application of pure and modified BN nanoclusters that has been widely explored in the literature is the use as a material for optical sensors.^26,58,76−79^ The UV–vis spectra were estimated using TD-DFT calculations with B3LYP/6-31g(d,p) to investigate the possibility of applying the Cu-modified B_12_N_12_ nanocage also as an optical sensor for Cad detection. The number of TD states in DFT was chosen to cover all vertical transitions from 150 to 650 nm. Figure 9 and Table 5 illustrate the UV–vis absorption spectra of pristine B_12_N_12_ and Cu(b_64_)B_12_N_12_ before and after the cadaverine interaction.

**Table 5:** Values of Adsorption Energy (*E*_ads_), Electronic Sensitivity (Δgap), in Addition to Sensitivity (*S*) and Selectivity Coefficient (κ) Calculated for the Interaction of Water and Gases NO, H_2_, H_2_S, CO, COCl_2_, N_2_O and N_2_ with Cu(b_64_)B_12_N_12_

system	*E*_gap_ (eV)	Δgap (%)	*S*	κ	*E*_ads_ (eV)	Φ (eV)	ΔΦ (%)	*S*_*j*_	κ_*j*_
Cu(b_64_)B_12_N_12_–Cad	4.25^α^	39.80	1.7 × 10^10^	1	–1.39	2.46	24.31	2.2 × 10^13^	1
Cu(b_64_)B_12_N_12_–NO	1.94^α^	36.18	2 × 10^9^	8.50	–1.25	3.37	3.69	1.1 × 10^2^	2.1 × 10^11^
Cu(b_64_)B_12_N_12_–H_2_O	4.09^α^	34.54	7.4 × 10^8^	22.5	–0.79	2.87	11.69	2.6 × 10^6^	8.5 × 10^6^
Cu(b_64_)B_12_N_12_–H_2_	4.03^α^	32.57	2.3 × 10^8^	72.2	–0.05	2.75	15.38	2.8 × 10^8^	8.0 × 10^4^
Cu(b_64_)B_12_N_12_–H_2_S	3.99^α^	31.25	1.1 × 10^8^	1.6 × 10^2^	–0.6	2.85	12.31	5.7 × 10^6^	3.9 × 10^6^
Cu(b_64_)B_12_N_12_–CO	2.48^α^	18.42	5.4 × 10^4^	3.1 × 10^5^	–1.09	3.25	0.00	0.00	–
Cu(b_64_)B_12_N_12_–COCl_2_	4.95^β^	18.42	3.2 × 10^6^	5.2 × 10^3^	–0.91	4.53	2.79	1.6 × 10^2^	1.4 × 10^11^
Cu(b_64_)B_12_N_12_–N_2_O	3.45^α^	13.49	2.9 × 10^3^	5.7 × 10^6^	–0.72	3.38	4.00	1.6 × 10^2^	1.4 × 10^11^
Cu(b_64_)B_12_N_12_–N_2_	2.78^α^	8.55	1.6 × 10^2^	1.1 × 10^8^	–0.48	3.50	7.69	1.7 × 10^4^	1.3 × 10^9^

The calculated data show that pure B_12_N_12_ absorbs most strongly at 198 nm, which is in agreement with previous experimental and theoretical studies.^60,78−82^ This peak corresponds to the excitation energy (*E* = 6.37 eV) and an oscillator strength (*f* = 0.036), with 39% contribution from the HOMO → LUMO transition and 35% from the H1 → L+1 transition. For pure Cu(b_64_)B_12_N_12_, the maximum absorbance peak (λ_max_) was observed at 250.8 nm, corresponding to an excitation energy (*E* = 4.8 eV) and an oscillator strength (*f* = 0.27), which is associated with the transition of an electron from the HOMO to the LUMO H(α) → L(α) (97%), as we have already observed in a previous study.^68^ With cadaverine adsorption, there was a splitting of the absorption band, resulting in two peaks with lower intensity: one in the 215 nm region, with *f* = 0.011 and *E* = 5.7 eV and the other blue-shifted at 350 nm, with *f* = 0.012 and energy *E* = 3.5 eV. These new peaks are associated with the H(α) → L(α) transitions of the system.

It is observed that modification of B_12_N_12_ with copper produces a shift of the absorption peak to regions of higher frequency. The adsorption of Cad on Cu(b_64_)B_12_N_12_ results in a shift of λ_max_ to the region closer to blue (350 nm). These results are promising for the use of copper-decorated boron nitride clusters in the development of optical sensors for cadaverine detection.

### Interferents and Selectivity Analysis

**Figure 10:**
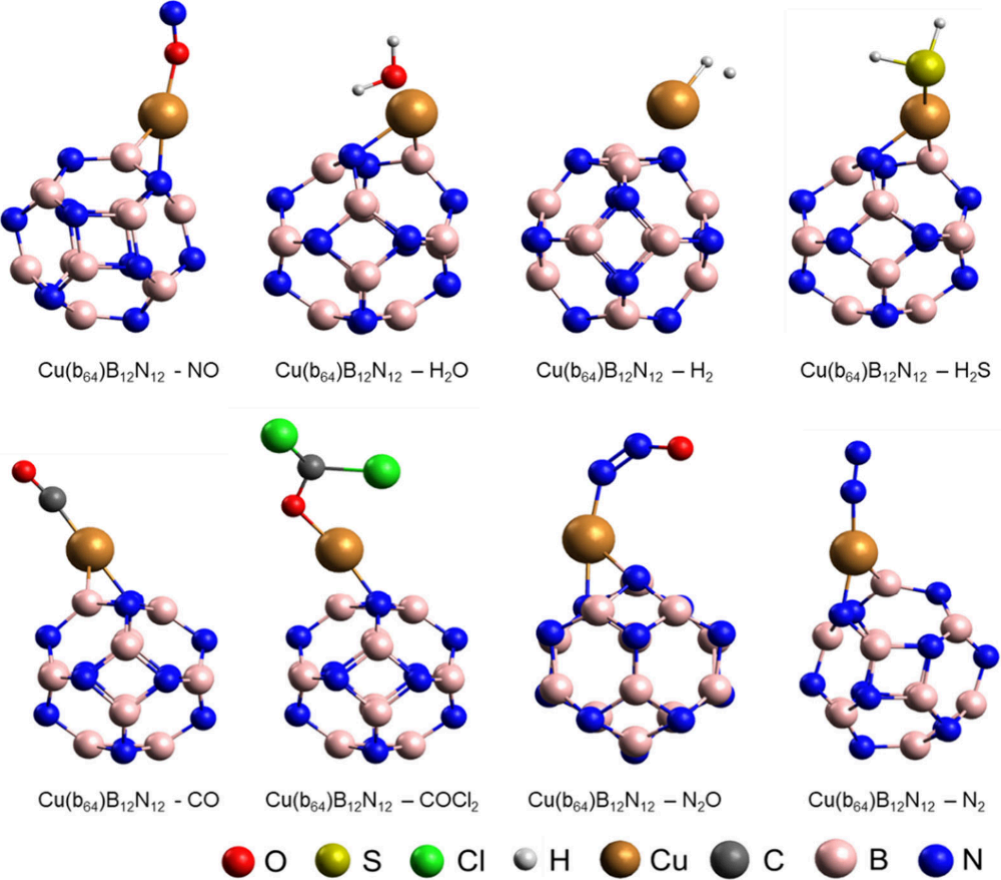
Optimized structures for the adsorption of H_2_O, NO, H_2_O, H_2_, H_2_S, CO, COCl_2_, N_2_O and N_2_ in Cu(b_64_)B_12_N_12_ nanocages.

Having presented better results for the interaction and detection of Cad, the system was subjected to interaction with water and the gases NO, H_2_O, H_2_, H_2_S, CO, COCl_2_, N_2_O and N_2_, to investigate its selectivity capacity. with sensor. The optimized structures of the interfering molecules adsorbed on the cage are shown in Figure 10.

The band gap (*E*_gap_), the sensitivity (Δgap), the adsorption energy (*E*_ads_), the work function (Φ), and the variation of the work function (ΔΦ), as well as the selectivity and the selectivity coefficient^83−86^ considering the sensitivity (*S* and κ) and the work function (*S*_*j*_ and κ_*j*_), were calculated for water and the gases NO, H_2_, H_2_S, CO, COCl_2_, N_2_O and N_2_ and then shown in Table 5.

It is possible to observe that the Cu(b_64_)B_12_N_12_ nanocage is more sensitive to Cad (Δgap = 39.80%) than to the other gases studied, indicating that Cad can be easily detected by the cage in a chimoresistive sensor, even in the presence of these gases or water. Furthermore, the Cu(b_64_)B_12_N_12_–Cad system presented the highest *E*_ads_ value in the series, i.e., more negative (−1.39 eV) and the greatest variation in the work function (ΔΦ = 24.31%), suggesting that the modified nanocage strongly adsorbs Cad and can be applied in electronic or work function sensors to detect diamine, even in the presence of interfering gases and in an aqueous or high humidity environment.

The selectivity (*S* and *S*_*j*_) adequately relates the electronic parameters to the proportional variation of the electrical properties of the sensor, that is, the variation in the adsorption energy gap (Δgap) and the variation in electrical conductivity (σ), variation of the function work (ΔΦ) and the change in electrical current density in the sensor (*j*), providing more-accurate information about the sensor’s ability to differentiate molecules. In addition, the selectivity coefficients (κ and κ_*j*_) indicate how many times the system is more sensitive to the molecule of interest in relation to the others.

Regarding electronic selectivity (*S*), it was observed that the Cu(b_64_)B_12_N_12_ nanocage can differentiate Cad from all interfering gases and even water, with a conductometric signal ten times greater for Cad (*S* = 1.7 × 10^10^), when compared with the best signal from the interferers (NO, *S* = 2 × 10^9^). The calculated selectivity coefficient (κ) indicates greater differentiation for the Cad/NO pair and lower differentiation for the Cad/N_2_ pair. It is worth mentioning that the higher the selectivity coefficient, the greater the differentiation between the molecules. Regarding the work function, the system is 10 000 times more selective to Cad (*S*_*j*_ = 2.2 × 10^13^) than to the best of the interferers (H_2_, *S*_*j*_ = 2.8 × 10^8^). It is observed that the adsorption of carbon monoxide (CO) on Cu(b_64_)B_12_N_12_ does not produce a change in the work function, which therefore presents zero selectivity for the gas. Furthermore, it appears that the selectivity coefficient for the work function has a higher value for Cad, compared with water and interferents. Finally, the results show that the Cu(b_64_)B_12_N_12_ nanocage is capable of simultaneously detecting Cad and NO gas in the environment and can selectively detect Cad in water and other gases analyzed.

### Humidity Analysis

**Figure 11:**
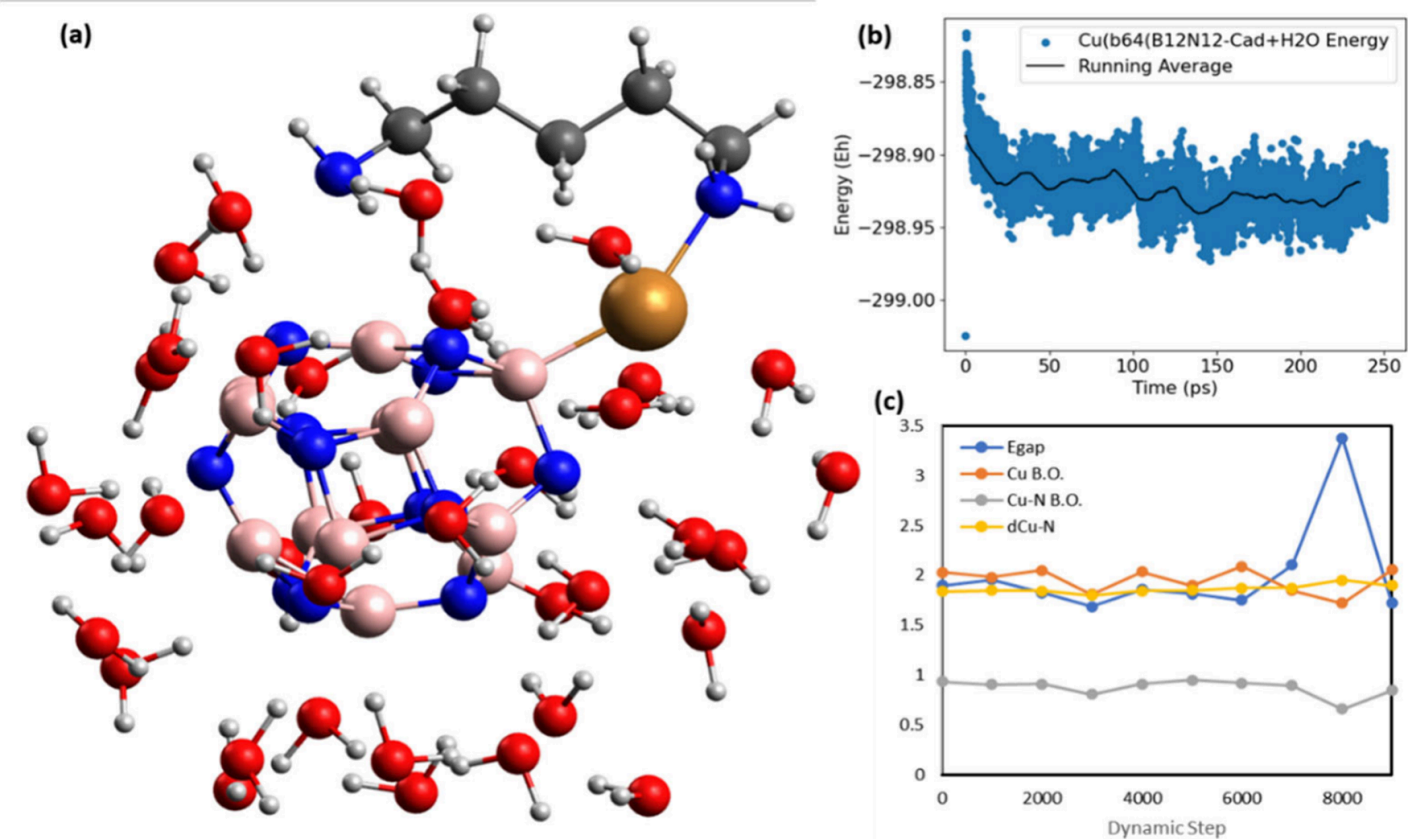
(a) Optimized structures of Cad adsorption on the Cu(b_64_)B_12_N_12_ nanocage surrounded by 40 H_2_O molecules, (b) molecular dynamics, and (c) *E*_gap_, number of Cu bonds, Cu–N bond order, and *d*_Cu–N_ bond distance calculated during MD.

For a better investigation of the influence of humidity on the cadaverine adsorption process on the surface of the Cu(b_64_)B_12_N_12_ nanocage, the Cad/cage system was inserted into a “water box” with 40 water molecules (Figure 11a), which simulates a concentration equivalent to that of the aqueous medium. The “water box” was subjected to quantum molecular dynamic (MD) with a period of 250 ps, with an integration time of 2 ps, to analyze the adsorption stability of Cad on the nanocage. During the dynamics, other parameters were monitored (Figure 11c), such as energy gap (*E*_gap_), bond number of the Cu atom, bond order and bond distance between copper and Cad nitrogen (Cu–N BO and *d*_Cu–N_, respectively). Figure 11b shows the energy variations of the system as a function of the processed dynamics time. It is observed that the energy of the system remains stable, with small variations, which are related to the movements and rearrangements of the structures. As seen in Figure 11a, Cad remains adsorbed on the surface of the cage throughout the entire time, even when surrounded by water, which indicates that the presence of water in the medium does not reduce the cage’s ability to adsorb the diamine molecule. Furthermore, the parameters calculated during MD (Figure 11c) remain constant, which corroborates the observed stability and confirms the ability of the nanocage to be used in sensors for detecting Cad also in liquid media.

## Conclusion

Cu-modified B_12_N_12_ and Al_12_N_12_ nanocages were investigated for cadaverine (Cad) detection. The Cu modification led to changes in geometry, an increase in dipole moment, and a reduction in the HOMO–LUMO gap of the nanocages. Cohesion energy (*E*_coh_) calculations and quantum descriptors indicated reduced stability after copper modification, and it was observed that the metal interacts better with B_12_N_12_ nanocage, while Cu-encapsulated nanocages showed lower cohesion. Pure B_12_N_12_ (Δgap = 2%) and Al_12_N_12_ (Δgap = 0.5%) nanocages were not sensitive to Cad adsorption, but the decorated Cu(b_64_)B_12_N_12_ and Cu(b_66_)Al_12_N_12_ showed higher electronic sensitivity to diamine (Δgap = 39.8% and 35.6%, respectively), surpassing the literature data. Adsorption energy (*E*_ads_) revealed a strong Cad/nanocage interaction (chemisorption), and the negative Δ*G*_ads_ confirmed spontaneous adsorption. However, molecular dynamics (MD) proved that the Cu(b_66_)Al_12_N_12_ nanocage is not stable, as it is converted to Cu(b_64_)Al_12_N_12_ during the process, making it unsuitable for sensing, despite its high sensitivity. Additionally, charge analysis indicated that the nanocages act as Lewis’s acids, extracting electrons from Cad.

The Cu(b_64_)B_12_N_12_–Cad system showed a high recovery time due to its elevated *E*_ads_, making it suitable for disposable sensor applications. But ultraviolet radiation and temperature can reduce Cad desorption time. UV–vis analysis showed that Cu(b_64_)B_12_N_12_ also optically responds to the presence of diamine, with a shift in the absorbance band. Furthermore, Cu(b_64_)B_12_N_12_ demonstrated high sensitivity and selectivity in detecting Cad in the presence of water and gases such as NO, H_2_, H_2_S, CO, COCl_2_, N_2_O, and N_2_. The Cu(b_64_)B_12_N_12_–Cad system was placed in a “box” with 40 water molecules and subjected to MD, which proved that adsorption is stable even under high humidity, which did not interfere with adsorption c Therefore, the results confirm that Cu(b_64_)B_12_N_12_ is promising for applications in electronic, optical, and work function sensors for selective cadaverine detection, even in high humidity environments. Future research may optimize the nanocages in other media, exploring different modification positions and concentrations of copper or other transition metals, aiming to maximize interaction with Cad. Its synthesis and testing as electrochemical sensors are steps toward the practical application of the material.

## Supporting Information Available

Input data of the molecular dynamics (MD) for B_12_N_12_ nanocage performed in xTB to investigate its stability and the partial density of states (PDOS) spectra indicate the changes in the electronic properties of Cu-modified B_12_N_12_ and Al_12_P_12_, before and after cadaverine adsorption (TXT)
